# Single Nucleotide Polymorphisms of Immunity-Related Genes and Their Effects on Immunophenotypes in Different Pig Breeds

**DOI:** 10.3390/genes12091377

**Published:** 2021-08-31

**Authors:** Ann Ying-An Chen, Chao-Wei Huang, Shyh-Hwa Liu, An-Chi Liu, Hso-Chi Chaung

**Affiliations:** 1 Research Center for Animal Biologics, National Pingtung University of Science and Technology, Neipu, Pingtung 912, Taiwan; noonoo452@hotmail.com (A.Y.-A.C.); realanns1x@gmail.com (A.-C.L.); 2 Department of Veterinary Medicine, National Pingtung University of Science and Technology, Neipu, Pingtung 912, Taiwan; 3 Department of Tropical Agriculture and International Cooperation, National Pingtung University of Science and Technology, Neipu, Pingtung 912, Taiwan; cwhuang@mail.npust.edu.tw; 4 Department of Animal Science, National Pingtung University of Science and Technology, Neipu, Pingtung 912, Taiwan; liush@mail.npust.edu.tw

**Keywords:** immune cells, immune-related genes, single-nucleotide polymorphism, pig

## Abstract

Enhancing resistance and tolerance to pathogens remains an important selection objective in the production of livestock animals. Single nucleotide polymorphisms (SNPs) vary gene expression at the transcriptional level, influencing an individual’s immune regulation and susceptibility to diseases. In this study, we investigated the distribution of SNP sites in immune-related genes and their correlations with cell surface markers of immune cells within purebred (Taiwan black, Duroc, Landrace and Yorkshire) and crossbred (Landrace-Yorkshire) pigs. Thirty-nine SNPs of immune-related genes, including 11 cytokines, 5 chemokines and 23 Toll-like receptors (TLRs) (interferon-α and γ (IFN-α, γ), tumor necrosis factor-α (TNF-α), granulocyte-macrophage colony-stimulating factor (GM-CSF), Monocyte chemoattractant protein-1 (MCP-1) and TLR3, TLR4, TLR7, TLR8, and TLR9) were selected, and the percentages of positive cells with five cell surface markers of CD4, CD8, CD80/86, MHCI, and MHCII were analyzed. There were 28 SNPs that were significantly different among breeds, particularly between Landrace and Taiwan black. For instance, the frequency of SNP1 IFN-α -235A/G in Taiwan black and Landrace was 11.11% and 96.15%, respectively. In addition, 18 SNPs significantly correlated with the expression of cell surface markers, including CD4, CD8, CD80/86, and MHCII. The percentage of CD4+ (39.27%) in SNP33 TLR-8 543C/C was significantly higher than those in A/C (24.34%), at *p* < 0.05. Together, our findings show that Taiwan black pigs had a unique genotype distribution, whereas Landrace and Yorkshire had a more similar genotype distribution. Thus, an understanding of the genetic uniqueness of each breed could help to identify functionally important SNPs in immunoregulation.

## 1. Introduction

In the past three decades, swine have become important in agricultural and biomedical research. Genetic improvements have been widely focused on production traits, such as growth and meat quality [1], or susceptibility to infectious pathogens [2]. The genomic sequence was released in 2012, which provided a valuable insight into swine immunology and biomedical research [3]. Identifying genetic polymorphisms of immunocompetent traits within the innate and adaptive immune system is essential for improving zootechnical performance and health in pigs [4]. These disease-resistant traits/genetic markers in pigs are extremely difficult to measure directly by pathogen exposure or challenge, due to genetic, environmental and virus mutation factors [4,5]. It is, however, possible to measure these immunity traits indirectly by means of phenotypic parameter analysis [4,6]. The induction of innate immune responses functions based on important receptors called pattern-recognition receptors (PRRs), and the most well-defined family is the Toll-like receptors (TLRs). TLRs are crucial in innate immunity for recognizing and the clearance of various infectious pathogens and the effectual establishment of acquired immunity by directly recognizing molecules from microbes [7].

To date, several TLRs have been linked to antiviral immunity, particularly TLR3, TLR7, TLR8, and TLR9. The initial engagement of TLRs with viral pathogen-associated molecular patterns (PAMPs; i.e., viral nucleic acids and viral proteins) is essential for the induction of the interferon (IFN)-mediated antiviral and inflammatory cytokine responses [8]. Indications of the immunoregulatory roles of type 1 IFN note an upregulation in the expression of MHC class I antigens [9]. It has been shown that IFNs can provide a costimulatory effect by binding to IFN1R on CD8 T cells and increasing proliferation [10], whereas exposure to IFN-γ promotes the differentiation of CD4 T cells [11]. T-cell activation not only requires antigen presentation by professional antigen-presenting cells (APC), but also costimulatory molecules provided by APC [12]. Monocyte chemoattractant protein-1 (MCP-1) plays an important role in the recruitment of monocytes and macrophages from the bloodstream to the inflamed tissue [13], whereas granulocyte-macrophage colony-stimulating factor (GM-CSF) appears to be a central factor for dendritic cell (DC) development [14] and also increases the expression of molecules involved in antigen presentation MHC class II and the costimulatory molecules CD80 and CD86 [15].

Single-nucleotide polymorphisms (SNPs) are genetic variations of single base-pairs occurring in the genomic sequence impacting gene expression activities, amino acid substitution, and dysfunction. SNPs occur in three different regions, in the promotor, extron and intron, and have been shown to impact gene expression at various degrees [16]. It was initially thought that non-coding SNPs did not possess functions in gene regulation, but recent studies show that these mutations can create alternative splicing sites to modulate protein diversity or exert a direct effect on the transcriptional level [17,18,19]. Notably, SNPs’ occurrence and frequency may be highly correlated with individuals’ susceptibility to pathogens and diseases and immunity responses [20]. Natural and artificial selection in pigs has shaped genetic adaptation due to environmental factors, and therefore they display significant phenotypic diversity [21]. The SNPs in human genomes leads to the alteration or enhancement of cytokine genes and protein structures, such as interleukin-1, interleukin-6, and interleukin-8 [22]. On the other hand, SNPs in the human TLR family also enhance the susceptibility or resistance to human pathogens and cancers; this suggests the importance of SNPs in immunity regulation [23]. There are ten TLR genes identified in the pig genome regarding the innate immunity system [24]. Sixty-three SNPs of TLR1, 2, 4, 5 and 6 were identified, causing amino acid substitution [25], and SNPs of TLR3, 7, and 8 have the potential to stimulate host immunity against a wide range of viral invasions, thus enhancing antiviral ability in different pig breeds [26].

An understanding of the genetic repertoire of each breed and their possible association with the immunophenotypes could help in identifying functionally important SNPs that regulate immune response. This could also be beneficial in establishing high pathogen-resistant breeding parameters which (**a**) enhance robustness, (**b**) reduce the use of antibiotics, (**c**) enhance vaccine responsiveness and (**d**) reduce economic losses to disease. The genetic analysis between Taiwan black, Duroc, Yorkshire and Landrace has already been defined [27]; however, knowledge as to how these breeds differ in immunological response and immunity traits is still insufficient. To begin to establish the differences in five common swine breeds in Taiwan, this study determined the SNPs of immune-related genes (IFN-α, IFN-γ, TNF-α, GM-CSF, MCP-1, TLR3, TLR4, TLR7, TLR8, and TLR9) and their correlation with positive cell surface markers of the immunophenotypes CD4, CD8, CD80/86, MHCI, and MHCII.

## 2. Materials and Methods

### 2.1. Sample Collection

Blood was collected from 187 four-week-old piglets of the breeds Taiwan black (*n* = 27), Duroc (*n* = 27), Landrace (*n* = 30), Yorkshire (*n* = 26), and Landrace–Yorkshire hybrid (*n* = 77), from three unrelated pig farms located in Pingtung, Taiwan. Samples were collected from the external jugular vein with a 21G needle in EDTA vacutainer tubes (Becton, Dickinson and Company (BD), Franklin Lakes, NJ, USA) and were brought back to the laboratory on ice for immediate analysis. All procedures were conducted with approval from the National Pingtung University of Science and Technology Institutional Animal Care and Use Community (IACUC) following AAALAC guidelines (IACUC protocol number NPUST-104-068).

### 2.2. Bioinformatics and Primer Design

The DNA sequence of the immune-related genes IFN-α, IFN-γ, TNF-α, GM-CSF, MCP-1, TLR3, TLR4, TLR7, TLR8, and TLR9 were obtained from the NCBI database. To identify these 39 SNPs, we initially screened over 56 sites in the promotor and various exon and intron regions, and confirmed these PCR fragments by sequencing. The sequenced fragments containing SNP sites were used to design PCR primers for the detection of SNP variance. We designed two methods for SNP detection: (**a**) restriction enzyme (RE) digestion (New England Biolabs Inc., Ipswich, MA, USA) for SNP sites located at certain restriction sites, and (**b**) amplification-refractory mutation system PCR (ARMS-PCR) for SNP sites without any restriction sites. Established for different purposes, 3′ end primers were designed for ARMS-PCR and two primers for RE digestion were used to identify the SNP sites. The locations of primer pairs are in the promoter or first exon region (listed in Table 1). All primers used in this study are listed in Supplemental Table S1.

### 2.3. Genomic DNA Isolation

To investigate the SNPs, 500 µL of whole blood was prepared for DNA extractions using the Genomic DNA Mini Kit for Blood & Cultured Cell (Geneaid, New Taipei City, Taiwan). All extractions were performed using the manufacturer’s protocol. DNA quality and concentration were quantified by a spectrophotometer with an A260/280 ratio of 1.8–2.0.

### 2.4. Amplification-Refractory Mutation System-Polymerase Chain Reaction and Restriction Enzyme Digestion

**Figure 1:**
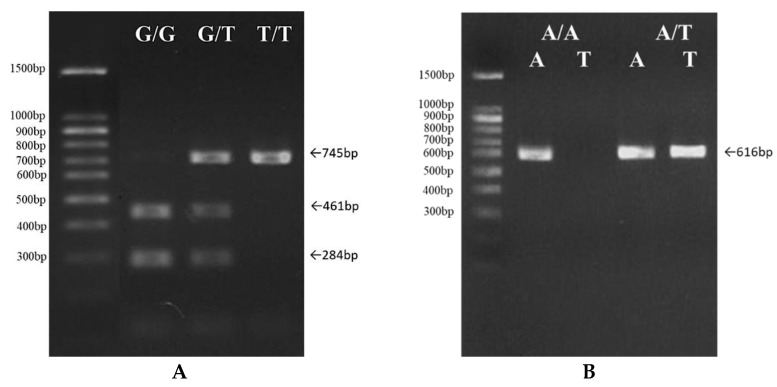
The detection of single nucleotide polymorphisms’ genotype distribution by ARMS-PCR. After the clean-up of PCR products, some were digested by RE, and PCR products were separated into different fragment sizes using 2% agarose gel electrophoresis to distinguish single-nucleotide variation. (**A**) Three genotypes, G/G, G/T, and T/T, in the SNP3 IFN-γ 490 gene. (**B**) Two genotypes, A/A and A/T, in the SNP19 TLR3 405 gene.

To detect mutations involving single base changes in immune-related genes, ARMS-PCR and restriction enzyme (RE) digestion were used. ARMS-PCR is based on using sequence-specific PCR primers to amplify target DNA that the nucleotide sequences contained in the sample. Following ARMS, the presence or absence of PCR products amplified from genomic DNA indicate single nucleotide variations (Figure 1). In parallel, some PCR products were digested by RE after clean up (RE listed in Table 1). Taq DNA Polymerase 2× Master Mix Red (Ampliqon, Odense M, Denmark) was used with the following PCR conditions: a pre-incubation for 15 min at 95 °C, 30 denaturation cycles at 95 °C for 30 s, annealing at 47–59 °C (temperatures listed in Table 1) for 40 s, extension at 72 °C for 30 s, and a final extension temperature of 60 °C for 5 min. The RE digested PCR product was separated into different fragments by using 2% agarose gel electrophoresis to distinguish single-nucleotide variation.

### 2.5. Isolation of PBMCs and Immunofluorescent Staining

**Figure 2:**
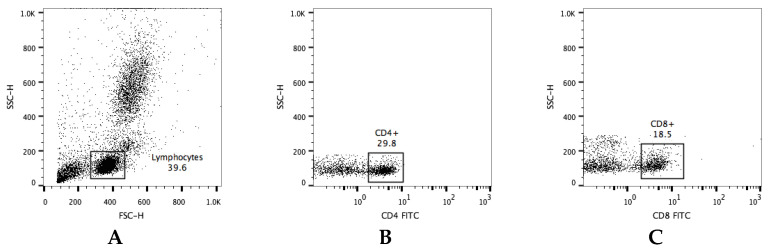

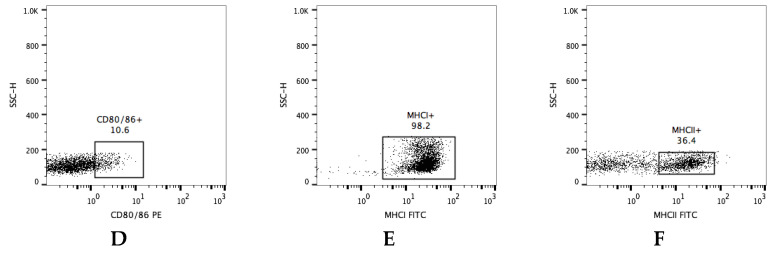
Gating strategy for flow cytometry data. Representative dot plots illustrating the gated (**A**) lymphocytes population within leukocytes. The gating strategy for the evaluation of lymphocyte subpopulations—(**B**) CD4+, (**C**) CD8+, (**D**) CD80/86+, (**E**) MHCI+, (**F**) MHCII+. The frequency of cells is expressed as a percentage of positive cells in a lymphocyte gate.

Peripheral blood mononuclear cells (PBMCs) were isolated from 10 mL of whole blood, and the samples were diluted at a ratio of 1:2 with phosphate-buffered saline (PBS; Lonza) containing 2% fetal bovine serum (FBS; Gibco, Thermo Fisher Scientific, Inc., Waltham, MA, USA), overlaid on Lymphoprep (Stemcell Technologies Inc., Vancouver, BC, Canada), and centrifuged at 800× *g* for 30 min. The PBMC layer was removed and transferred into ammonium-chloride-potassium (ACK) lysing buffer (Gibco) for the lysis of red blood cells. PBMCs were washed twice with RPMI-1640 medium (FBS 10%, penicillin 100 IU/mL and streptomycin 0.1 mg/mL, Gibco), the cells were adjusted to 1 × 10^6^ cells/mL with PBS and separated into 9 light-protected 1.5 mL microcentrifuge tubes, each containing 1 × 10^5^ cells/mL. The tubes were centrifuged, the supernatant was discarded, and the pellet was resuspended with 2% bovine serum albumin (BSA) in PBS. The cells were incubated for 30 min at 4 °C with mAb against surface molecules (see below). After staining, the cells were washed twice with 1mL of PBS and fixed with 4% paraformaldehyde for 30 min at 4 °C. Lastly, the cells were resuspended with 1% BSA/PBS and stored at 4 °C for later testing with an EPICS^®^ XL-MCL flow cytometer (Beckman Coulter). The flow cytometry gates were set on singlets, following lymphocytes and live cells (SSC-H and FSC-H). All gating strategies for immune cells are shown in Figure 2.

### 2.6. Antibodies

Specific primary and isotype control antibodies were used in this study. Isotype-matched unspecific antibodies served as a negative control. Fluorochrome-conjugated primary mAbs included: mouse CD4-fluorescein (FITC) (clone 74-12-4, Abcam, CB, Bristol, UK), mouse CD8-fluorescein (FITC) (clone 76-2-11, Abcam), CD80/86 (fusion protein Human CTLA-4)-phycoerythrin (PE) (clone BHK, Ancell, Stillwater, MN, USA), mouse anti-pig MHCI-fluorescein (FITC) (clone JM1E3, Bio-Rad, Hercules, CA, USA) and mouse anti-pig MHCII-fluorescein (FITC) (clone 2E9/13, Bio-Rad, Hercules, CA, USA). Unspecific binding of primary antibodies that were not directly conjugated with fluorochromes was evaluated by means of the use of mouse IgG1-fluorescein (FITC) (Bio-Rad), mouse IgG2a-fluorescein (FITC) (clone MOPC-173, Biolegend, San Diego, CA, USA), mouse IgG2b-phycoerythrin (PE) (clone MG2b-57, Biolegend) and mouse IgG2b-fluorescein (FITC) (Bio-Rad).

### 2.7. Statistical Analysis

Flow cytometric data were analyzed with FlowJo software (Tree Star, San Carlos, CA, USA). Data were analyzed using SAS software’s (Version 9.1.2, SAS Institute Inc., Cary, NC, USA) GLM procedures. Tukey’s honest significant difference test was used for comparisons if a significant F static was detected by ANOVA. Observed genotypes were used to estimate allele frequencies for each SNP by chi-square distribution, and departures from Hardy–Weinberg equilibrium were assessed using Pearson’s goodness-of-fit test. *p*-values less than 0.05 indicated that the results were significantly different.

## 3. Results

### 3.1. Genotype Distribution of Single Nucleotide Polymorphisms in Cytokine Genes in Association with Different Pig Breeds and Correlation with Their Immunophenotypes

There were eleven SNP sites of cytokine genes analyzed in this study; all indicated significant differences among breeds except for SNP9: GM-CSF (741). The genotype distribution and allele frequencies of the eleven SNPs of cytokine genes were investigated in relation to different pig breeds (Table 2) and their correlation with their immunophenotypes (Table 3). The allele frequencies in all five pig groups of SNPs in cytokine genes did not differ significantly between each genotype in univariate analysis. A higher prevalence of the promotor region SNP1: IFN-α-235A/G genotype was observed in the Duroc, Landrace, and Yorkshire pig breeds (*p* < 0.0001). SNP1: IFN-α-235A/G polymorphism was associated with a higher CD4:CD8 ratio and a higher percentage of MHCII-positive cells. A predominantly higher frequency of SNP2: IFN-γ 382C/T genotype was noted in the Taiwan black and Landrace–Yorkshire breeds (*p* < 0.0001), and this variant showed a positive correlation with a higher expression of CD4+ cells. Among the pig breeds, Taiwan black had the highest distribution in the SNP6: TNF-α 1219A/A genotype and showed a correlation with increased expression of CD8+ and MHCII cells. Moreover, the SNP8: GM-CSF 245C/T showed a correlation with increased CD4+ and CD8+; interestingly, this genotype was predominant in the Taiwan black, Landrace, and Landrace–Yorkshire breeds.

### 3.2. Genotype Distribution of Single Nucleotide Polymorphisms in Chemokines Genes in Association with Different Pig Breeds and Correlation with Their Immunophenotypes

There were five SNP sites in the first intron of MCP-1 gene analyzed in this study, three SNP12: MCP-1 (273), SNP13: MCP-1 (336), and SNP14: MCP-1 (351) were not significantly different among the pig breeds. The genotype distribution and allele frequencies of the two SNPs of chemokine genes were investigated in relation to different pig breeds (Table 4) and their correlation with their immunophenotypes (Table 5). The allele frequencies in all five pig groups of SNPs in chemokine genes did not differ significantly amongst each genotype in univariate analysis. A significantly higher prevalence of the SNP15: MCP 360T/T genotype was observed in the Duroc, Landrace, Yorkshire, and Landrace–Yorkshire pig breeds (*p* < 0.0001). In addition, the SNP15: MCP-1 360C/T gene polymorphism was associated with higher levels of CD4+, CD8+, and MHCII cells. Furthermore, there was a more predominant frequency of the SNP16: MCP 383A/A genotype observed in the Taiwan black, Landrace, Yorkshire, and Landrace–Yorkshire pig breeds.

### 3.3. Genotype Distribution of Single Nucleotide Polymorphisms in Toll-Like Receptor Genes in Association with Different Pig Breeds and Correlation with Immunophenotypes

Ten of the twenty-three SNPs in the Toll-like receptor genes analyzed in this study indicated an association between the pig breeds. The genotype distribution and allele frequencies of the ten SNPs of Toll-like receptor genes were investigated in relation to different pig breeds (Table 6) and their correlation with their immunophenotypes (Table 7). The allele frequencies in all five pig groups of SNPs in TLR genes did not differ significantly amongst each genotype in univariate analysis. A higher prevalence of the SNP17: TLR3 95G/G and SNP20: TLR3 800C/T genotypes were observed in the Duroc, Landrace, Yorkshire, and Landrace–Yorkshire groups (*p* < 0.0001); interestingly, the SNP17: TLR3 95G/G polymorphism was associated with a higher CD4:CD8 ratio. Additionally, 357G/G, 1413T/T, and 2034A/A TLR7 gene polymorphism were more predominant in Taiwan black. A predominantly higher frequency of SNP34: TLR8 534C/C genotype was noted in Landrace–Yorkshire (*p* < 0.0001), and this variant showed a correlation with a higher expression of CD4+ and CD8+ cells. Additionally, SNP39: TLR9 1186C/T genotype was observed in all pig breeds.

## 4. Discussion

This study investigated 39 SNPs of immune-related gene variation between common pig breeds in Taiwan and sought to determine whether there were any correlations between the genotype and phenotype. The 39 SNPs’ genotype distribution was analyzed for 187 pigs via ARMS-PCR with or without restrictive enzyme digestion, and the phenotypes were analyzed by flow cytometry to observe CD4-, CD8-, CD80/86-, MHCI-, and MHCII-positive cell surface markers. In a previous study by Wilkie et al., it was indicated that immune response varies within different pig breeds [2]. Methods for improving genetic resistance in pigs have been used to control infectious diseases, and have been shown to be effective in salmonella-infected pigs [28,29]. Zhang et al. summarized the genomic diversity within different pig populations and noted that Asian breeds have considerably more variability than European breeds, supporting the documented history of pig breed domestication [30]. One study by Bergman et al. showed that genetic variations of TLRs, including SNPs, indicated differences between wild boars and domestic pigs [31]. Thus, this study is the first, to our knowledge, to analyze SNP genotype distribution in immune genes against immune cell surface markers in several important swine breeds.

Our results indicate that Taiwan black had distinctive genotype variations compared to the other pig breeds. The frequency of five cytokine SNP genes—the SNP1:IFN-α-235G/G, SNP2:IFN-γ 382C/T, SNP5:TNF-α 755T/T, SNP6:TNF-α 1219A/A, and SNP11:GM-CSF 782C/C genotypes—were all significantly higher in Taiwan black pig breeds; these genes were also associated with a higher CD4+ and CD8+ T cell population. Interestingly, the genotype distribution for all SNPs of cytokine genes in Taiwan black were consistent with the Hardy–Weinberg equilibrium (*p* > 0.05), except for SNP1:IFN-α(-235) and SNP2:IFN-γ (382). In a study by Huang et al., they analyzed eight IFN-γ SNP regions in humans, and suggested that the promotor region of the −764G/C IFN-γ gene is functionally important in determining viral clearance and treatment response in hepatitis C virus-infected patients [32]. It has also been indicated that eliciting two different phenotypes of cytotoxic T-lymphocytes, CD4+CD8- and CD4-CD8+, and neutralizing antibodies could be key factors in controlling porcine reproductive and respiratory syndrome virus (PPRSV) infection [33]. Another study demonstrated that CD4-CD8+ T lymphocytes are essential traits in disease resistance and could mediate the activity of CSFV-specific cytotoxic T lymphocytes [34]. Therefore, these genotypes and their association with higher levels of CD4+ and CD8+ T cells could be attributed to the host being genetically resistant to infectious diseases.

Taiwan black and Duroc had a slightly comparable genotype variation frequency in cytokine genes compared to other breeds. This may be the result of Taiwan black being a synthetic line, generated by the crossbreeding of Taoyuan and Duroc breeds [35]. They had similar SNP distribution in five cytokine genes, SNP4:TNF-α 366A/A, SNP5:TNF-α 755T/T, SNP6:TNF-α 1219A/G, SNP7:GM-CSF 193T/T, and SNP11:GM-CSF 782C/C. Interestingly, the SNP4:TNF-α 366A/A and SNP7:GM-CSF 193T/T gene polymorphisms were associated with a higher percentage of MHC class II-positive cells within lymphocytes. It has been reported that TNF-α promotes dendritic cell differentiation [36,37]. A study by Hornell et al. also identified a pathway by which GM-CSF activated APC function, where GM-CSF specifically induced types I and III CIITA, ultimately leading to increased MHC class II expression [38]. Interestingly, we see an upregulation in MHC class II molecules in Taiwan black and Duroc breeds. We noted that SNP15:MCP-1 360C/C genotype polymorphism was significantly higher in Taiwan black pigs, whereas Duroc, Landrace and Yorkshire shared a more similar genotype distribution of MCP-1 360T/T. In addition, the MCP-1 360C/C and C/T gene polymorphisms were both associated with a higher percentage of CD8 positive cells (Table 5). A previous study demonstrated that a genetic polymorphism of MCP-1 -362CC genotype contributed to the protection of pulmonary tuberculosis in human patients in Ghana [39]. Polymorphisms in MCP-1 could play a significant role in the migration, generation, and survival of memory and effector CD8+ T cells [40].

It is worth noting that TLR gene polymorphisms were more consistent throughout all five pig breeds. Additionally, the Duroc, Landrace, and Yorkshire breeds exerted a similar pattern in genotype distribution. The variations of five TLR genes, SNP17:TLR3 95A/A, SNP20: TLR3 800T/T, SNP27:TLR7 2034A/A, SNP35 TLR8 570A/A, and SNP39:TLR9 1186C/C, were all significantly higher in Taiwan black, whereas in the other breeds they had a fairly similar genotype distribution. We did not see any association between the TLR genotypes and their immunophenotypes. Morozumi and Shinkai et al. noted that in pigs, the nonsynonymous RNA-sensing TLR genes such as TLR3, 7, 8, and 9 presented fewer polymorphisms causing amino acid changes in cell-surface genes [26,41]; this proposes a similar pattern in humans. This could possibly explain why we were unable to see a correlation between these TLR genes and phenotypes. A predominantly higher frequency of the SNP34: TLR8 534C/C genotype was noted in Landrace–Yorkshire (*p* < 0.0001), and this variant showed a correlation with a higher expression of CD4+ and CD8+ cells. Ligation of multiple TLRs concurrently or in sequence, particularly TLR3 or TLR4 together with TLR7, TLR8, or TLR9, has been shown to induce a synergistic increase in the production of multiple cytokines produced by dendritic cells [41]. Moreover, a total of 139 nonsynonymous SNPs in the coding and non-coding regions of cattle in TLR3, TLR7, and TLR8 were identified [42].

Disease association can be seen in functional single nucleotide polymorphisms (SNPs) (i.e., those that affect gene expression, mRNA stability, or protein structure). Our results demonstrate the SNP distribution that exists in common pig breeds in Taiwan, and these include cytokines, chemokines, and TLR genes. Numerous polymorphisms were identified within the coding and non-coding regions of cytokines and several disease-associated studies based on these variants. Bidwell et al. summarized several reviews regarding these associated studies and created an online database [20,43,44]. A review also discussed different cell surface and extracellular TLR polymorphisms and their association and susceptibility to infectious diseases in humans [45].

Furthermore, we noted that Landrace and Yorkshire had a more similar SNP distribution, especially in IFN-α, IFN-γ, and TNF-α cytokine genes. Interestingly, the SNP analyses in TLR genes suggest that Duroc, Landrace, and Yorkshire had similar genotype distribution and were quite different from Taiwan black. Several observations have indicated connections between polymorphisms in cytokines, chemokines, or pattern recognition receptor-related genes and disease susceptibility [22,26,46,47]. Chen’s study confirmed that Taiwan black pigs are phylogenetically more related to European breeds than Chinese breeds [27]. However, some contradictory results were present in our dataset; importantly, our study found that Taiwan black pigs are unique compared to European pig breeds according to SNP and immunity parameter analysis. It is also possible that the offspring with disease-related characteristics were selected through crossbreeding, and the majority of genotype distributions were inherited from Chinese pig breeds rather than European pig breeds. In future, it will be important to investigate the genotype distribution of SNPs in Taoyuan pigs, which may help explain these differences between Taiwan black pigs from different Asian sources.

Ultimately, there is still insufficient research conducted in pigs which demonstrate SNPs’ association with diseases and the host’s susceptibility to infectious disease. We noted that a higher frequency of mutations occurred in cytokine and chemokine polymorphisms, and that TLR genes indicated fewer genetic variations within the different breeds. More importantly, SNP genetic variations of immune-related genes did occur within common pig breeds in Taiwan. Our findings are an example of applying candidate gene polymorphism approaches to identifying functionally important mutations that may affect immunophenotype expressions which could influence host susceptibility to infectious pathogens, but further studies are required to better understand the underlying correlation between these genotypes and immunophenotypes, and could potentially be used as an indirect method for measuring immune traits. If true, SNPs in immune-related genes could potentially to be used for breed selection of pigs which are more resistant to infectious pathogens. In future studies, we aim to expand our breed and sample count, investigate whether these SNPs follow a genetic hitchhiking pattern in related pigs, and integrate different detection methods such as real-time PCR. However, despite advances in SNP genotyping with next-generation sequencing (NGS) [48], ARMS-PCR has still proven to be a simple, fast, and inexpensive method for detecting single nucleotide polymorphisms. Nevertheless, these results could potentially aid in predicting immune response phenotypes in animals’ pre-vaccination and influence the design of better vaccines through the generation of new knowledge and the identification of targets and biomarkers for vaccine response.

## Figures and Tables

**Table 1:** The PCR analytic conditions and their production.

SNP No.	SNPs	PCR Annealing (°C)	PCR Product (bp)	Restriction Enzyme
SNP1	IFN-α (-235)	54	212	*
SNP2	IFN-γ (382)	53	507	*
SNP3	IFN-γ (490)	50	836	SspI
SNP4	TNF-α (366)	54	352	BtsCI
SNP5	TNF-α (755)	53	174	*
SNP6	TNF-α (1219)	59	550	*
SNP7	GM-CSF (193)	53	1046	ApaLI
SNP8	GM-CSF (245)	55	437	BccI
SNP9	GM-CSF (741)	53	421	*
SNP10	GM-CSF (753)	53	1046	BsoBI
SNP11	GM-CSF (782)	54	469	TseI
SNP12	MCP-1 (273)	54	252	*
SNP13	MCP-1 (336)	52	443	BtsI
SNP14	MCP-1 (351)	52	443	Bsp1286I
SNP15	MCP-1 (360)	52	443	MseI
SNP16	MCP-1 (383)	52	443	BtsCI
SNP17	TLR 3 (95)	53	926	*
SNP18	TLR 3 (159)	55	857	*
SNP19	TLR 3 (405)	55	616	*
SNP20	TLR 3 (800)	53	221	*
SNP21	TLR 4 (-13)	54	406	*
SNP22	TLR 7 (-332)	47	331	*
SNP23	TLR 7 (66)	55	562	*
SNP24	TLR 7 (357)	55	272	*
SNP25	TLR 7 (1413)	59	899	*
SNP26	TLR 7 (1633)	55	677	*
SNP27	TLR 7 (2034)	55	346	*
SNP28	TLR 7 (22,996)	47	459	*
SNP29	TLR 8 (14)	47	739	*
SNP30	TLR 8 (41)	55	1029	*
SNP31	TLR 8 (124)	52	628	*
SNP32	TLR 8 (176)	55	577	*
SNP33	TLR 8 (265)	56	807	*
SNP34	TLR 8 (534)	56	402	*
SNP35	TLR 8 (570)	59	377	*
SNP36	TLR 9 (872)	55	681	*
SNP37	TLR 9 (905)	55	653	MSPA1I
SNP38	TLR 9 (1126)	57	506	*
SNP39	TLR 9 (1186)	55	367	*

*: SNPs were confirmed by using ARMS-PCR without restriction of enzyme digestion.

**Table 2:** The genotype distribution of SNPs in cytokine genes and their association with different pig breeds.

Cytokine Polymorphism	Genotype Frequencies among Breed					
Genotype	Taiwan Black *n* (%)	Duroc *n* (%)	Landrace *n* (%)	Yorkshire *n* (%)	Landrace Yorkshire *n* (%)	*p*-Value ^a^
SNP1: IFN-α (−235)	*n* = 27	*n* = 27	*n* = 30	*n* = 26	*n* = 77	
A/A	10 (37.04)	0	2 (6.67)	0	22 (28.57)	
A/G	3 (11.11)	20 (74.07)	26 (86.67)	25 (96.15)	35 (45.45)	<0.0001
G/G	14 (51.85)	7 (25.93)	2 (6.67)	1 (3.85)	20 (25.97)	
Allele frequency *p*-value ^b^	0.57	0.63	0.5	0.51	0.49	
Genotype distribution *p*-value ^c^	<0.0001	<0.0001	<0.0001	<0.0001	<0.0001	
HWE *p*-value ^d^	<0.0001	0.002	<0.0001	<0.0001	0.428	
SNP2: IFN-γ (382)	*n* = 27	*n* = 27	*n* = 30	*n* = 26	*n* = 77	
C/C	3 (11.1)	12 (44.44)	10 (33.33)	1 (3.85)	29 (37.66)	
C/T	23 (85.2)	15 (55.56)	17 (56.67)	12 (46.15)	48 (62.34)	<0.0001
T/T	1 (3.7)	0	3 (10)	13 (50)	0	
Allele frequency *p*-value ^b^	0.46	0.28	0.38	0.73	0.28	
Genotype distribution *p*-value ^c^	<0.0001	<0.0001	<0.0001	<0.0001	<0.0001	
HWE *p*-value ^d^	0.0002	0.456	0.276	0.378	0.001	
SNP3: IFN-γ (490)	*n* = 24	*n* = 27	*n* = 30	*n* = 26	*n* = 77	
G/G	9 (37.5)	0	12 (40)	10 (38.46)	6 (7.89)	
G/T	9 (37.5)	13 (48.15)	15 (50)	14 (53.85)	47 (61.84)	<0.0001
T/T	6 (25)	14 (51.85)	3 (10)	2 (7.69)	23 (30.26)	
Allele frequency *p*-value ^b^	0.44	0.76	0.35	0.35	0.61	
Genotype distribution *p*-value ^c^	<0.0001	<0.0001	<0.0001	<0.0001	<0.0001	
HWE *p*-value ^d^	0.243	0.099	0.588	0.333	0.008	
SNP4: TNF-α (366)	*n* = 24	*n* = 25	*n* = 30	*n* = 26	*n* = 75	
A/A	12 (50)	12 (48)	0	0	17 (22.67)	
A/G	9 (37.5)	3 (12)	2 (6.67)	3 (11.54)	7 (9.33)	<0.0001
G/G	3 (12.5)	10 (40)	28 (93.33)	23 (88.46)	51 (68)	
Allele frequency *p*-value ^b^	0.31	0.46	0.97	0.94	0.73	
Genotype distribution *p*-value ^c^	<0.0001	<0.0001	<0.0001	<0.0001	<0.0001	
HWE *p*-value ^d^	0.532	<0.0001	0.85	0.754	<0.0001	
SNP5: TNF-α (755)	*n* = 27	*n* = 27	*n* = 30	*n* = 26	*n* = 77	
C/C	3 (11.11)	1 (3.7)	12 (40)	12 (46.15)	13 (16.88)	
C/T	10 (37.04)	16 (59.26)	16 (53.33)	13 (50)	62 (80.52)	<0.0001
T/T	14 (51.85)	10 (37.04)	2 (6.67)	1 (3.85)	2 (2.6)	
Allele frequency *p*-value ^b^	0.7	0.67	0.33	0.29	0.43	
Genotype distribution *p*-value ^c^	<0.0001	<0.0001	<0.0001	<0.0001	<0.0001	
HWE *p*-value ^d^	0.561	0.083	0.273	0.266	<0.0001	
SNP6: TNF-α (1219)	*n* = 26	*n* = 27	*n* = 30	*n* = 26	*n* = 77	
A/A	10 (38.46)	0	0	0	2 (2.6)	
A/G	15 (57.69)	26 (96.3)	7 (23.33)	6 (23.08)	67 (87.01)	<0.0001
G/G	1 (3.85)	1 (3.7)	23 (76.67)	20 (76.92)	8 (10.39)	
Allele frequency *p*-value ^b^	0.33	0.52	0.88	0.88	0.54	
Genotype distribution *p*-value ^c^	<0.0001	<0.0001	<0.0001	<0.0001	<0.0001	
HWE *p*-value ^d^	0.112	<0.0001	0.469	0.505	<0.0001	
SNP7: GM-CSF (193)	*n* = 21	*n* = 27	*n* = 30	*n* = 25	*n* = 76	
C/C	0	0	11 (36.67)	3 (12)	4 (5.63)	
C/T	1 (4.76)	9 (33.33)	14 (46.67)	8 (32)	35 (49.3)	<0.0001
T/T	20 (95.24)	18 (66.67)	5 (16.67)	14 (56)	32 (45.07)	
Allele frequency *p*-value ^b^	0.98	0.83	0.4	0.72	0.7	
Genotype distribution *p*-value ^c^	<0.0001	<0.0001	<0.0001	<0.0001	<0.0001	
HWE *p*-value ^d^	0.911	0.29	0.87	0.302	0.158	
SNP8: GM-CSF (245)	*n* = 19	*n* = 27	*n* = 30	*n* = 26	*n* = 45	
C/C	2 (10.53)	4 (14.81)	0	1 (3.85)	1 (2.22)	
C/T	10 (52.63)	4 (14.81)	22 (73.33)	1(3.85)	26 (57.78)	<0.0001
T/T	7 (36.84)	19 (70.37)	8 (26.67)	24 (92.31)	18 (40)	
Allele frequency *p*-value ^b^	0.63	0.78	0.63	0.94	0.69	
Genotype distribution *p*-value ^c^	<0.0001	<0.0001	<0.0001	<0.0001	<0.0001	
HWE *p*-value ^d^	0.568	0.002	0.001	0.0009	0.019	
SNP10: GM-CSF (753)	*n* = 21	*n* = 27	*n* = 30	*n* = 26	n = 47	
C/C	3 (14.29)	6 (22.22)	7 (23.33)	3 (11.54)	27 (57.45)	
C/T	9 (42.86)	16(59.26)	19 (63.33)	23 (88.46)	0	<0.0001
T/T	9 (42.86)	5(18.52)	4 (13.33)	0	20 (42.55)	
Allele frequency *p*-value ^b^	0.64	0.48	0.45	0.44	0.43	
Genotype distribution *p*-value ^c^	<0.0001	<0.0001	<0.0001	<0.0001	<0.0001	
HWE *p*-value ^d^	0.759	0.427	0.125	<0.0001	<0.0001	
SNP11: GM-CSF (782)	*n* = 23	*n* = 27	*n* = 30	*n* = 26	*n* = 75	
C/C	20 (86.96)	17 (62.96)	6 (20)	14 (53.85)	22 (29.33)	
C/T	3 (13.04)	10 (37.04)	14 (46.67)	10 (38.46)	35 (46.67)	<0.0001
T/T	0	0	10 (33.33)	2 (7.69)	18 (24)	
Allele frequency *p*-value ^b^	0.07	0.19	0.57	0.27	0.47	
Genotype distribution *p*-value ^c^	<0.0001	<0.0001	<0.0001	<0.0001	<0.0001	
HWE *p*-value ^d^	0.737	0.237	0.785	0.908	0.579	

HWE, Hardy–Weinberg equilibrium; GM-CSF, granulocyte-macrophage colony-stimulating factor; ^a^: χ2-test: different genotype distribution among the five different pig breeds, ^b^: χ2-test: allele frequency among the three genotype groups, ^c^: χ2-test: genotype distribution within each pig group, ^d^: HWE test, *p* values less than 0.05 were not consistent with HWE.

**Table 3:** Effects of SNPs in cytokine genes on the expressions of immunity parameters in pig lymphocytes.

Cytokine Polymorphism	Immunity Parameters (%)	Genotype		
		1	2	3
SNP1: IFN-α(-235)		A/A	A/G	G/G
	CD8	25.68 ± 3.22 ^a^	20.27 ± 1.18 ^a^	32.86 ± 2.42 ^b^
	CD4:CD8 ratio	1.39 ± 0.22 ^ab^	1.66 ± 0.14 ^a^	1.07 ± 0.09 ^b^
	MHCII	23.79 ± 2.45 ^a^	31.86 ± 1.32 ^b^	23.75 ± 1.95 ^a^
SNP2: IFN-γ (382)		C/C	C/T	T/T
	CD4	27.96 ± 2.61 ^a^	26.8 ± 1.22 ^a^	17.72 ± 2.30 ^b^
SNP3: IFN-γ (490)		G/G	G/T	T/T
	CD8	28.55 ± 2.79 ^a^	47.43 ± 4.5 ^b^	22.9 ± 1.95 ^a^
	MHCII	33.21 ± 2.48 ^a^	55.88 ± 4.18 ^b^	24.06 ± 1.76 ^c^
SNP4: TNF-α (366)		A/A	A/G	G/G
	CD8	26.13 ± 2.87 ^a^	28.57 ± 3.33 ^a^	22.24 ± 1.25 ^b^
	MHCII	29.06 ± 2.23 ^a^	23.07 ± 2.26 ^b^	29.83 ± 1.39 ^a^
SNP5: TNF-α (755)		C/C	C/T	T/T
	CD4	29.19 ± 3.01 ^a^	26.79 ± 1.29 ^ab^	20.31 ± 1.75 ^a^
	CD8	26.8 ± 1.98 ^a^	20.99 ± 1.28 ^b^	34.05 ± 3.29
	CD4:CD8 ratio	1.2 ± 0.14 ^a^	1.73 ± 0.13 ^b^	0.74 ± 0.09 ^a^
	MHCII	36.68 ± 2.20 ^a^	25.55 ± 1.25 ^b^	32.29 ± 2.38 ^a^
SNP6: TNF-α (1219)		A/A	A/G	G/G
	CD8	41.04 ± 3.22 ^a^	21.34 ± 1.35 ^b^	25.85 ± 1.88 ^b^
	CD4:CD8 ratio	0.65 ± 0.12	1.6 ± 0.11	1.42 ± 0.21
	MHCII	31.83 ± 2.62 ^a^	25.14 ± 1.25 ^b^	35.64 ± 1.86 ^a^
SNP8: GM-CSF (245)		C/C	C/T	T/T
	CD4	21.31 ± 4.08 ^a^	30.75 ± 2.06 ^b^	23.36 ± 1.74 ^a^
	CD8	19.58 ± 3.62 ^a^	29.41 ± 2.07 ^b^	22.35 ± 1.30 ^a^
	MHCII	14.23 ± 4.28 ^a^	30.14 ± 1.93 ^b^	31.19 ± 1.63 ^b^
SNP10: GM-CSF (753)		C/C	C/T	T/T
	CD4	31.78 ± 2.29 ^a^	24.13 ± 1.88 ^b^	23.81 ± 2.34 ^b^
	MHCII	21.07 ± 2.29 ^a^	35.9 ± 1.52 ^b^	29.15 ± 2.84 ^b^
SNP11: GM-CSF (782)		C/C	C/T	T/T
	CD8	25.86 ± 1.85 ^a^	24.29 ± 1.60 ^a^	18.26 ± 2.81 ^b^
	CD4:CD8 ratio	1.23 ± 0.10 ^a^	1.47 ± 0.16 ^a^	2.18 ± 0.33 ^b^

Means with different letters in superscript (a, b, and c) are significantly different (*p* < 0.05).

**Table 4:** The genotype distribution of SNPs in chemokine genes and their association with different pig breeds.

Chemokine Polymorphism	Genotype Frequencies among Breed					
Genotype	Taiwan Black *n* (%)	Duroc *n* (%)	Landrace *n* (%)	Yorkshire *n* (%)	Landrace Yorkshire *n* (%)	*p*-Value ^a^
SNP15: MCP-1 (360)	*n* = 27	*n* = 27	*n* = 30	*n* = 26	*n* = 77	
C/C	25 (92.59)	6 (22.22)	7 (23.33)	3 (11.54)	27 (35.06)	
C/T	2 (7.41)	1 (3.7)	3 (10)	0	0	<0.0001
T/T	0	20 (74.07)	20 (66.67)	23 (88.46)	50 (64.94)	
Allele frequency *p*-value ^b^	0.04	0.76	0.72	0.88	0.65	
Genotype distribution *p*-value ^c^	<0.0001	<0.0001	<0.0001	<0.0001	<0.0001	
HWE *p*-value ^d^	0.841	<0.0001	<0.0001	<0.0001	<0.0001	
SNP16: MCP-1 (383)	*n* = 27	*n* = 27	n = 30	*n* = 26	*n* = 77	
A/A	23 (85.19)	16 (59.26)	27 (90)	21 (80.77)	77 (100)	
A/G	2 (7.41)	10 (37.04)	0	5 (19.23)	0	<0.0001
G/G	2 (7.41)	1 (3.7)	3 (10)	0	0	
Allele frequency *p*-value ^b^	0.11	0.22	0.1	0.1	-	
Genotype distribution *p*-value ^c^	<0.0001	<0.0001	<0.0001	<0.0001	<0.0001	
HWE *p*-value ^d^	0.001	0.71	<0.0001	0.587	-	

HWE, Hardy–Weinberg equilibrium; MCP-1, monocyte chemoattractant protein-1 ^a^: χ2-test: different genotype distribution among the five different pig breeds, ^b^: χ2-test: allele frequency among the three genotype groups, ^c^: χ2-test: genotype distribution within each pig group, ^d^: HWE test, *p* values less than 0.05 were not consistent with HWE.

**Table 5:** Effects of SNPs in chemokine genes on the expression of immunity parameters in pig lymphocytes.

Chemokine Polymorphism	Immune Parameter (%)	Genotype		
		1	2	3
SNP13: MCP-1 (336)		A/A	A/C	C/C
	MHCII	7.45 ± 0.55 ^a^	28.49 ± 1.01 ^b^	38.48 ± 6.75 ^c^
SNP15: MCP-1 (360)		C/C	C/T	T/T
	CD4	28.92 ± 1.70 ^ab^	32.86 ± 9.92 ^a^	23.98 ± 1.31 ^a^
	CD8	31.26 ± 2.07 ^a^	32.46 ± 6.49 ^a^	18.92 ± 1.00 ^b^
	CD4:CD8 ratio	1.18 ± 0.09 ^a^	1.11 ± 0.23 ^a^	1.7 ± 0.15 ^b^
	MHCII	23.97 ± 1.59 ^a^	39.7 ± 11.81 ^b^	31.33 ±1.19 ^b^
SNP16:MCP-1 (383)		A/A	A/G	G/G
	CD8	24.66 ± 1.22 ^a^	16.94 ± 1.71 ^b^	32.46 ± 6.49 ^c^

Means with different letters in superscript (a, b, and c) are significantly different (*p* < 0.05).

**Table 6:** The genotype distribution of SNPs in TLR genes and their association with different pig breeds.

TLR Polymorphism	Genotype Frequencies among Breed					
Genotype	Taiwan Black *n* (%)	Duroc *n* (%)	Landrace *n* (%)	Yorkshire *n* (%)	Landrace Yorkshire *n* (%)	*p*-Value ^a^
SNP17: TLR3 (95)	*n* = 25	*n* = 26	*n* = 30	*n* = 26	*n* = 77	
A/A	10 (40)	4 (15.38)	10 (33.33)	0	5 (6.49)	
A/G	4 (16)	1 (3.85)	0	0	4 (5.19)	<0.0001
G/G	11 (44)	21 (80.77)	20 (66.67)	26 (100)	68 (88.31)	
Allele frequency *p*-value ^b^	0.52	0.83	0.67	1	0.91	
Genotype distribution *p*-value ^c^	<0.0001	<0.0001	<0.0001	<0.0001	<0.0001	
HWE *p*-value ^d^	0.0006	<0.0001	<0.0001	<0.0001	<0.0001	
SNP20: TLR3 (800)	*n* = 27	*n* = 26	*n* = 30	*n* = 26	*n* = 77	
C/T	8 (29.63)	25 (96.15)	29 (96.67)	25 (96.15)	69 (89.61)	<0.0001
T/T	19 (70.37)	1 (3.85)	1 (3.33)	1 (3.85)	8 (10.39)	
Allele frequency *p*-value ^b^	0.85	0.52	0.52	0.52	0.55	
Genotype distribution *p*-value ^c^	<0.0001	<0.0001	<0.0001	<0.0001	<0.0001	
HWE *p*-value ^d^	0.366	<0.0001	<0.0001	<0.0001	<0.0001	
SNP23: TLR7 (66)	*n* = 27	*n* = 26	*n* = 30	*n* = 13	*n* = 77	
C/T	3 (11.11)	0	4 (13.33)	1 (3.85)	32 (41.56)	<0.0001
T/T	24 (88.89)	26 (100)	26 (86.67)	12 (96.15)	45 (58.44)	
Allele frequency *p*-value ^b^	0.94	1	0.93	0.96	0.79	
Genotype distribution *p*-value ^c^	<0.0001	<0.0001	<0.0001	<0.0001	<0.0001	
HWE *p*-value ^d^	0.759	-	0.695	0.885	0.021	
SNP24: TLR7 (357)	*n* = 26	*n* = 27	*n* = 29	*n* = 26	*n* = 77	
A/G	12 (46.15)	22 (84.62)	20 (68.97)	13 (50)	67 (87.01)	<0.0001
G/G	14 (53.85)	4 (15.38)	9 (31.03)	13 (50)	10 (12.99)	
Allele frequency *p*-value ^b^	0.77	0.58	0.66	0.75	0.56	
Genotype distribution *p*-value ^c^	<0.0001	<0.0001	<0.0001	<0.0001	<0.0001	
HWE *p*-value ^d^	0.126	<0.0001	0.004	0.08	<0.0001	
SNP25: TLR7 (1413)	*n* = 23	*n* = 26	*n* = 30	*n* = 27	*n* = 77	
C/T	2 (8.7)	13 (50)	25 (83.33)	13 (46.15)	35 (45.45)	<0.0001
T/T	21 (91.3)	13 (50)	5 (16.67)	14 (53.85)	42 (54.55)	
Allele frequency *p*-value ^b^	0.96	0.75	0.58	0.76	0.77	
Genotype distribution *p*-value ^c^	<0.0001	<0.0001	<0.0001	<0.0001	<0.0001	
HWE *p*-value ^d^	0.827	0.089	<0.0001	0.099	0.009	
SNP27: TLR7 (2034)	*n* = 27	*n* = 26	*n* = 29	*n* = 26	*n* = 77	
A/A	11 (40.74)	0	0	1 (3.85)	0	<0.0001
A/G	16 (59.26)	26 (100)	29 (100)	25 (96.15)	77 (100)	
Allele frequency *p*-value ^b^	0.3	0.5	0.5	0.48	0.5	
Genotype distribution *p*-value ^c^	<0.0001	<0.0001	<0.0001	<0.0001	<0.0001	
HWE *p*-value ^d^	0.028	<0.0001	<0.0001	<0.0001	<0.0001	
SNP30: TLR8 (41)	*n* = 27	*n* = 26	*n* = 30	*n* = 26	*n* = 77	
G/G	0	0	1 (3.33)	0	3 (3.9)	
G/T	7 (25.93)	0	11 (36.67)	24 (92.31)	29 (37.66)	<0.0001
T/T	20 (74.07)	26 (100)	18 (60)	2 (7.69)	45 (58.44)	
Allele frequency *p*-value ^b^	0.87	1	0.78	0.54	0.77	
Genotype distribution *p*-value ^c^	<0.0001	<0.0001	<0.0001	<0.0001	<0.0001	
HWE *p*-value ^d^	0.438	-	0.66	<0.0001	0.525	
SNP34: TLR8 (534)	*n* = 27	*n* = 26	*n* = 30	*n* = 26	*n* = 77	
A/A	1 (3.7)	0	0	0	1 (1.3)	
A/C	26 (96.3)	26 (100)	30 (100)	26 (100)	52 (67.53)	<0.0001
C/C	0	0	0	0	24 (31.17)	
Allele frequency *p*-value ^b^	0.48	0.5	0.5	0.5	0.65	
Genotype distribution *p*-value ^c^	<0.0001	<0.0001	<0.0001	<0.0001	<0.0001	
HWE *p*-value ^d^	<0.0001	<0.0001	<0.0001	<0.0001	<0.0001	
SNP35: TLR8 (570)	*n* = 27	*n* = 27	*n* = 30	*n* = 26	*n* = 77	
A/A	14 (51.85)	0	0	0	1 (1.3)	<0.0001
A/T	13 (48.15)	26 (100)	30 (100)	26 (100)	76 (98.7)	
Allele frequency *p*-value ^b^	0.24	0.5	0.5	0.5	0.49	
Genotype distribution *p*-value ^c^	<0.0001	<0.0001	<0.0001	<0.0001	<0.0001	
HWE *p*-value ^d^	0.099	<0.0001	<0.0001	<0.0001	<0.0001	
SNP39: TLR9 (1186)	*n* = 27	*n* = 27	*n* = 30	*n* = 26	*n* = 27	
C/C	7 (25.93)	0	0	0	0	<0.0001
C/T	20 (74.07)	27 (100)	30 (100)	26 (100)	27 (100)	
Allele frequency *p*-value ^b^	0.37	0.5	0.5	0.5	0.5	
Genotype distribution *p*-value ^c^	<0.0001	<0.0001	<0.0001	<0.0001	<0.0001	
HWE *p*-value ^d^	0.002	<0.0001	<0.0001	<0.0001	<0.0001	

HWE, Hardy–Weinberg equilibrium; TLR, Toll-like receptor ^a^: χ2-test: different genotype distribution among the five different pig breeds, ^b^: χ2-test: allele frequency among the three genotype groups, ^c^: χ2-test: genotype distribution within each pig group, ^d^: HWE test, *p* values less than 0.05 were not consistent with HWE.

**Table 7:** Effects of SNPs in Toll-like receptor genes on the expressions of immunity parameters in pig lymphocytes.

TLR Polymorphism	Immune Parameter (%)	Genotype		
		1	2	3
SNP17: TLR 3 (95)		A/A	A/G	G/G
	CD8	28.25 ± 2.66 ^ab^	33.29 ± 6.00 ^a^	22.44 ± 1.22 ^b^
	CD4:CD8 ratio	1.02 ± 0.12 ^a^	0.9 ± 0.16 ^a^	1.63 ± 0.12 ^b^
SNP18: TLR 3 (159)		C/C	C/T	T/T
	CD4:CD8 ratio	1.64 ± 0.21 ^a^	1.82 ± 0.23 ^a^	1.28 ± 0.07 ^b^
SNP21: TLR 4 (-13)		A/A	A/G	G/G
	CD8	15.85 ± 1.54 ^a^	22.46 ± 1.36 ^a^	15.64 ± 1.32 ^a^
	MHCII	23.31 ± 1.82 ^a^	37.23 ± 1.49 ^b^	17.59 ± 1.45 ^c^
SNP34: TLR 8 (534)		A/A	A/C	C/C
	CD4	36.25 ± 11.05 ^a^	24.34 ± 1.00 ^b^	39.27 ± 4.18 ^a^
	CD8	32.85 ± 11.05 ^a^	22.53 ± 1.11 ^b^	35.65 ± 3.77 ^a^

Means with different letters in superscript (a, b, and c) are significantly different (*p* < 0.05).

## Supplementary Materials

The following are available online at https://www.mdpi.com/article/10.3390/genes12091377/s1, Table S1: Summary of primer sequences used for ARMS-PCR in discovering SNP immune-related genes.

## Abbreviations

ACK, ammonium-chloride-potassium; ARMS-PCR, amplification-refractory mutation system PCR; BSA, bovine serum albumin; CD, cluster of differentiation; DNA, deoxyribonucleic acid; FITC, fluorescein isothiocyanate; GM-CSF, granulocyte-macrophage colony-stimulating factor; HWE, Hardy–Weinberg equilibrium; IFN, interferon (e.g., IFN-α,γ); MCP, monocyte chemoattractant protein; MHC, major histocompatibility complex; PBMC, peripheral blood mononuclear cell; PBS, phosphate-buffered saline; PE, phycoerythrin; RE, restriction enzyme; RPMI, Roswell Park Memorial Institute; SNP, single-nucleotide polymorphism; TLR, Toll-like receptor; TNF, tumor necrosis factor.
