# Supplementary material for: Single Nucleotide Polymorphisms of Immunity-Related Genes and Their Effects on Immunophenotypes in Different Pig Breeds
Source: Genes (Basel). 2021 Aug 31;12(9):1377. doi: 10.3390/genes12091377 (PMC8471458; doi:10.3390/genes12091377)
Supplement: Supplementary file 1 [file genes-12-01377-s001.zip › genes-1334561-supplementary.pdf]

Supplementary Table I. Summary of primer sequences used for ARMS-PCR in discovering SNPs in cytokine, chemokine and toll-like receptor immune-related genes.

| Primers | SNPs Genetic Locus | Accession No. | Phenotype | Sequence (from 5´to 3´) | Source |
|---|---|---|---|---|---|
| SNP 1 | IFN-α (-235) | X57191 | Promoter | F: ACCTCATGGTTCCTAGTCGTATTC<br>R(A): TCTTTTTCATTATTGCCTTCCGTT<br>R(G): TCTTTTTCATTATTGCCTTCCGTC | Designed |
| SNP 2 | IFN-γ (382) | X53085 | Intron 1 | F(C): AGCATAGTATGGGTATAAATCGTC<br>F(T): AGCATAGTATGGGTATAAATCGTT<br>R: TTCTATTACCTCTACCTATGTCC | Designed |
| SNP 3 | IFN-γ (490) | X53085 | Intron 1 | F: TCAGCTTTGCGTGACTTTG<br>R: TTCTATTACCTCTACCTATGTCC | Designed |
| SNP 4 | TNF-α (366) | NC_010449 | Intron 1 | F: TCTGCCTACTGCACTTCGAGG<br>R: CTATCTTTCTACCACACCCCT | Designed |
| SNP 5 | TNF-α (755) | NC_010449 | Intron 1 | F(T): GGACTTATTCAAATCTAATTAATCCTT<br>F(C): GGACTTATTCAAATCTAATTAATCCTC<br>R: AATTTCCCTTCTTCCTTCCAC | Designed |
| SNP 6 | TNF-α (1219) | NC_010449 | Intron 3 | F: AAGCTAGAGAAGCAGGTGGCTG<br>R(A): ATCCCATCTGTCCATGAGGTTCT<br>R(G):ATCCCATCTGTCCATGAGGTTCC | Designed |
| SNP 7 | GM-CSF (193) | U67318 | Intron 1 | F: TGTGGATGCCATCAAAGAAGC<br>R: AGTGCTGCTCATAGTGCTTGG | Designed |
| SNP 8 | GM-CSF (245) | U67318 | Intron 1 | F: TGTGGATGCCATCAAAGAAGC<br>R: ATCCCCTGGTGACTCCTGG | Designed |
| SNP 9 | GM-CSF (741) | U67318 | Intron 1 | F(C): AAGGAGAGTAACTGCCTTTCC<br>F(G): AAGGAGAGTAACTGCCTTTCG<br>R: AGTGCTGCTCATAGTGCTTGG | Designed |
| SNP 10 | GM-CSF (753) | U67318 | Intron 1 | F: TGTGGATGCCATCAAAGAAGC<br>R: AGTGCTGCTCATAGTGCTTGG | Designed |
| SNP 11 | GM-CSF (782) | U67318 | Intron 1 | F: TGGGCAGTCCTAGCATTCAAG<br>R: TCATTTCTTCCTGTTCTCATCC | Designed |
| SNP 12 | MCP-1 (273) | CU928660 | Intron 1 | F: ACTGCAGCCACCTTCTG<br>R(T): GCTGGGGTCCTGGGAA<br>R(C): GCTGGGGTCCTGGGAG | Designed |
| SNP 13 | MCP-1 (336) | CU928660 | Intron 1 | F: TTAACTGCACGATCAGGAA<br>R: ACTGCCTAACTTCTGGGAGAG | Designed |
| SNP 14 | MCP-1 (351) | CU928660 | Intron 1 | F: TTAACTGCACGATCAGGAA<br>R: ACTGCCTAACTTCTGGGAGAG | Designed |
| SNP 15 | MCP-1 (360) | CU928660 | Intron 1 | F: TTAACTGCACGATCAGGAA<br>R: ACTGCCTAACTTCTGGGAGAG | Designed |
| SNP 16 | MCP-1 (383) | CU928660 | Intron 1 | F: TTAACTGCACGATCAGGAA<br>R: ACTGCCTAACTTCTGGGAGAG | Designed |
| SNP 17 | TLR 3 (95) | NC_010457 | Exon 3 | F(A): ACCAACAAATGTACTGTTCA<br>F(G): ACCAACAAATGTACTGTTCG<br>R: TCCTCTTACCAAGATAAAAG | Designed |
| SNP 18 | TLR 3 (159) | NC_010457 | Exon 3 | F(C):GACCTCCCGGCAAAC<br>F(T):GACCTCCCGGCAAAT<br>R: TCCTCTTACCAAGATAAAAG | Designed |
| SNP 19 | TLR 3 (405) | NC_010457 | Exon 3 | F(A):TCCATCTAATGTCCAACTCA<br>F(T):TCCATCTAATGTCCAACTCT<br>R: TCCTCTTACCAAGATAAAAG | Designed |
| SNP 20 | TLR 3 (800) | NC_010457 | Exon 3 | F(C): TTCATGAACAGCAACATTCC<br>F(T): TTCATGAACAGCAACATTCT<br>R: TCCTCTTACCAAGATAAAAG | Designed |
| SNP 21 | TLR 4 (-13) | AY753179 | Promoter | F: TGGAAAGCCACCGTCC<br>R (A): GAATCATCCTGGCATTTGCT<br>R (G): GAATCATCCTGGCATTTGCC | Designed |
| SNP 22 | TLR 7 (-332) | AB291813 | Intron 1 | F(A): TTTTGAGAATCTAAAGCGA<br>F(G): TTTTGAGAATCTAAAGCGG<br>R: TCAAACAGAGAAGCCTGG | Designed |
| SNP 23 | TLR 7 (66) | AB291813 | Intron 1 | F(C): TTGAGGAAGAACTGAAACC<br>F(T): TTGAGGAAGAACTGAAACT<br>R: TAGTAATTTTCCATCCACTGA | Designed |
| SNP 24 | TLR 7 (357) | AB291813 | Intron 1 | F(A): GTGCTTTTTCTAGTGTAGGA<br>F(G): GTGCTTTTTCTAGTGTAGGG<br>R: TAGTAATTTTCCATCCACTGA | Designed |
| SNP 25 | TLR 7 (1413) | AB291813 | Intron 1 | F(C): GTTGTGCTAAAGACTGGATGTCTCC<br>F(T): GTTGTGCTAAAGACTGGATGTCTCT<br>R: TGCACCCAAGGCACATGGA | Designed |
| SNP 26 | TLR 7 (1633) | AB291813 | Intron 1 | F(C): AAGTTGGGATATCTTTTTTTAAC<br>F(T): AAGTTGGGATATCTTTTTTTAAT<br>R: TGCACCCAAGGCACATGGA | Designed |
| SNP 27 | TLR 7 (2034) | AB291813 | Intron 1 | F(A): GATGTGGGAACTCCACTTCAAA<br>F(G): GATGTGGGAACTCCACTTCAAG<br>R: ATATAGCAGGATCTCATTGCTTATC | Designed |
| SNP 28 | TLR 7 (22996) | AB291813 | Exon 3 | F(A): TAAATTTTTGCATCTTCTTCA<br>F(C): TAAATTTTTGCATCTTCTTCC<br>R: AAGGAGGAAGAAGGCTC | Designed |
| SNP 29 | TLR 8 (14) | AB291813 | Exon 1 | F(T): GAAAACATGACCCTTCACAT<br>F(G): GAAAACATGACCCTTCACAG<br>R: CGCTTAGATCCAGTACTCTTA | Designed |
| SNP 30 | TLR 8 (41) | AB291813 | Exon 1 | F(T): CTGACCTGCCTTTTCCTGCT<br>F(G): CTGACCTGCCTTTTCCTGCG<br>R: GGAAATATTAATGTACTGGGGATA | Designed |
| SNP 31 | TLR 8 (124) | AB291813 | Exon 1 | F(C): AGAAAAGAAAATGGCTCTC<br>F(G): AGAAAAGAAAATGGCTCTG<br>R: CGCTTAGATCCAGTACTCTTA | Designed |
| SNP 32 | TLR 8 (176) | AB291813 | Exon 1 | F(A): GAAGTACCCCGAAGAGTGTA<br>F(G): GAAGTACCCCGAAGAGTGTG<br>R: CGCTTAGATCCAGTACTCTTA | Designed |
| SNP 33 | TLR 8 (265) | AB291813 | Exon 1 | F(A): GGGCTGCAAAATCTGACTAAAA<br>F(G): GGGCTGCAAAATCTGACTAAAG<br>R: GGAAATATTAATGTACTGGGGATA | Designed |
| SNP 34 | TLR 8 (534) | AB291813 | Exon 1 | F(A): TGGAACTGCTATTTTACTTGTAATGAA<br>F(C): TGGAACTGCTATTTTACTTGTAATGAC<br>R: CAAATGATGCAGATTCTCAAACCAA | Designed |
| SNP 35 | TLR 8 (570) | AB291813 | Exon 1 | F(A): ATAGACGAGGGAGCATTTGAAAATTTA<br>F(T): ATAGACGAGGGAGCATTTGAAAATTTT<br>R: TGCAGCACCTTCAAATGATGCAGA | Designed |
| SNP 36 | TLR 9 (872) | CU915558 | Intron 1 | F(A): GGTGGAGTTGTGGCCATGTA<br>F(G): GGTGGAGTTGTGGCCATGTG<br>R: GCACCTCCGTGAGATTGTTGTA | Designed |
| SNP 37 | TLR 9 (905) | CU915558 | Intron 1 | F: TCAATGAACGCTCTAGGT<br>R:TGTAGCTCAGGTTCAGCTC | Designed |
| SNP 38 | TLR 9 (1126) | CU915558 | Intron 1 | F: TCAATGAACGCTCTAGGT<br>R(A): CGAAGTCGGAGTCGT<br>R(G): CGAAGTCGGAGTCGC | Designed |
| SNP 39 | TLR 9 (1186) | CU915558 | Intron 1 | F(C): AACCTCAAGTGGAACTGCCC<br>F(T): AACCTCAAGTGGAACTGCCT<br>R: GCACCTCCGTGAGATTGTTGTA | Designed |
